# Supplementary material for: Patterns of Intron Gain and Loss in Fungi
Source: PLoS Biol. 2004 Nov 30;2(12):e422. doi: 10.1371/journal.pbio.0020422 (PMC532390; doi:10.1371/journal.pbio.0020422)
Supplement: Table S1 — Also available at http://genes.mit.edu/NielsenEtAl/. (4.3 MB ZIP). [file pbio.0020422.st001.zip › NielsenEtAl/html/489.html]

AN1726.1.NCU09864.1.MG03840.1.FG09240.1


```
 CLUSTAL W (1.82) Multiple Sequence Alignments - Introns Inserted


Sequence 1: MG03840.1	463 aa
Sequence 2: FG09240.1	449 aa
Sequence 3: NCU09864.1	395 aa
Sequence 4: AN1726.1	464 aa
Alignment Length: 464 aa
Number Identitical Residues: 218 aa
Alignment Score (without introns) 11296


MG03840.1 	MRVRLPGAASSIGRRCRNHGRGAPKIGSLHQTAPGSASSMRAASTLKQKPDSSF~VSFPG
NCU09864.1	------------------------------------------------------~-----
FG09240.1 	MKSR---ALHQVALRR--------AILSRPHVTP---LAIRSASSVSQRPNSNF~VSFPG
AN1726.1  	MTPLIYLPGRVRSAHAVRCSLLKSPLQHPFRSSYPLSLHKRWSTSLSQRPGSDR2VRFPG
          	       .    .      .   .        :   :     ::: .. ..:.     ..

MG03840.1 	AVKSEFTTKFQFERPEQYPAMPTYRVVDQHGVVVDPE-FEQDLNDEGVIKLYEDMVAV~S
NCU09864.1	---------MTFEEPSKYDALPTYRAVDQHGQVVDPS-FKPDLSDKEVIKLYHDMLTV~S
FG09240.1 	ALKSAFTSNLKFESPESYPALSTYRVVDQHGVVVDES-FKPDISNEEVIRLYKDMVFI~S
AN1726.1  	AVNSKFTTEMAFINPMDKPGIPTYRVMDSDGVLIDKSRSELSVSNEEALAWYRNMLTV1S
          	: .:  ::.: *  * .  .:.***.:*..* ::* .  : .:.:: .:  *.:*: : *

MG03840.1 	VMDSIMFDAQRQGRVSFYM~VSAGEEAVCVGSASALEMRDVIFCQYREQGVYRHRGMTFR
NCU09864.1	IMDLIMFDAQRQGRLSFYM~VSAGEEAVCVGSASALSPEDVVFCQYREQGVFKQRGFQLS
FG09240.1 	IMDLIMFDAQRQGRLSFYM~VSAGEEAVSIGSSSVLDREDVMFTQYREQGVFKERGFTAK
AN1726.1  	IMDVIMFEAQRQGRLSFYM0VSAGEEGISVGSAAALTPDDVVFAQYRETGVFQQRGFALK
          	:** ***:******:**** ******.:.:**::.*   **:* **** **::.**:   

MG03840.1 	DFMNQLFANKYDPGQGRNMPVHYGSKELNM0HTISSPLATQIPQASGAAYAMKMQRIANP
NCU09864.1	DFMNQLFANHKDSGKGRNMPVHYGSKELNI0HTISSPLATQLPQAAGAAYALKIQRMQNP
FG09240.1 	DFMGQLFGNVRDPSRGRSMPVHYGSKELNI0HSVSSPLATQLPHASGAAYALKMQKLQNP
AN1726.1  	NFMSQLFANANDNGRGRNMPVHYGCEYPKT0HTISSTLATQIPQASGAAYALKLQALQNP
          	:**.***.*  * .:**.******.:  :  *::**.****:*:*:*****:*:* : **

MG03840.1 	DAPERVAVVYFGEGAASEGDFHAALNIAATRACPVIFICRNNGYSISTPSLEQYKGDGIA
NCU09864.1	TVPPRVVAAYFGEGAASEGDFHAALNIAATRGCPAVFICRNNGYAISTPTLEQYRGDGIA
FG09240.1 	SSKARVAVAYFGEGAASEGDFHAALNIAATRACPAIFICRNNGYAISTPTLDQYRGDGIA
AN1726.1  	DTPPRIVACYFGEGAASEGDFHAGLNIAATRSCPVVFICRNNGYAISTPTLEQYRGDGIA
          	    *:.. **************.*******.**.:********:****:*:**:*****

MG03840.1 	SRGVGYGIDTVRVDGNDIWAVRKATKKARELALENGGKPVLLECLTYRVGHHSTSDDSFA
NCU09864.1	SRGLGYGIETIRVDGNDFWAVREVTKRARELALQDGGKPVLIEAMTYRVSHHSTSDDSFA
FG09240.1 	SRGIGYGIDTIRVDGNDIWAVREVVKKAREMALEDGGKPILIEAMTYRVSHHSTSDDSFA
AN1726.1  	SRGVGYGIDTIRVDGNDIFAVYEAMKAARTLALSQGGKPVLIEAMSYRVSHHSTSDDSFA
          	***:****:*:******::** :. * ** :**.:****:*:*.::***.**********

MG03840.1 	YRARVEVEDWKRRDNPISRLRKWMESKDMWDDDKEKVMRDRLRKEILKAFSEAEKEKKPP
NCU09864.1	YRAKVEVEDWKRRDNPIGRLRKWMEAKGIWDENMEREARDSIRRDILKAFSQAEKEKKPA
FG09240.1 	YRARVEVEDWKRRDNPITRLRKWMEAQGIWDEAKEKEARTDLRKEILKAFSEAEREKKPP
AN1726.1  	YRARVEVEDWKRRDNPIIRLRKWLENEGIWNEDMERQARESIRKEVLREFGEAERAKKPA
          	***:************* *****:* :.:*::  *:  *  :*:::*: *.:**: ***.

MG03840.1 	IRAMFEDVYEEMTPDLKKQMAELKDHIERYPEEYDVGEFEGGKDSLKP
NCU09864.1	IRTMFEDVYEEMTPEIKAQMKELKEHLEQYPEEYDLGDFEGGKDSLAR
FG09240.1 	MRSMFEDMYEEMTDDLKAQMKELKEMLDKYPEEYDVSEFEGGKDTLKP
AN1726.1  	IRFAFEDVYDEVTEEAREQMKELRRILEEYPEEYDLRSFEGGVKGLDS
          	:*  ***:*:*:* : : ** **:  ::.******: .**** . *
```
